# Orthopaedic Surgery Elicits a Systemic Anti-Inflammatory Signature

**DOI:** 10.3390/jcm9072123

**Published:** 2020-07-06

**Authors:** Cortney E. Heim, Kelsey J. Yamada, Rachel Fallet, Jessica Odvody, Dana M. Schwarz, Elizabeth R. Lyden, Matthew J. Anderson, Roxanne Alter, Debbie Vidlak, Curtis W. Hartman, Beau S. Konigsberg, Chris A. Cornett, Kevin L. Garvin, Naglaa Mohamed, Annaliesa S. Anderson, Tammy Kielian

**Affiliations:** 1 Department of Pathology and Microbiology, University of Nebraska Medical Center, Omaha, NE 68198, USA; cortney.heim@unmc.edu (C.E.H.); kelsey.yamada@unmc.edu (K.J.Y.); rwfallet@unmc.edu (R.F.); jessica.odvody@unmc.edu (J.O.); ralter@unmc.edu (R.A.); dvidlak@unmc.edu (D.V.); 2 Orthopaedic Surgery and Rehabilitation, University of Nebraska Medical Center, Omaha, NE 68198, USA; dschwarz@unmc.edu (D.M.S.); cwhartma@unmc.edu (C.W.H.); bkonigsb@unmc.edu (B.S.K.); chris.cornett@unmc.edu (C.A.C.); kgarvin@unmc.edu (K.L.G.); 3 Department of Biostatistics, University of Nebraska Medical Center, Omaha, NE 68198, USA; elyden@unmc.edu; 4 College of Public Health Research Design and Analysis, University of Nebraska Medical Center, Omaha, NE 68198, USA; mandersonj@unmc.edu; 5 Bacterial Vaccines, Pfizer Vaccine Research and Development, Pearl River, NY 10965, USA; Naglaa.Mohamed@pfizer.com (N.M.); Annaliesa.anderson@pfizer.com (A.S.A.)

**Keywords:** arthroplasty, myeloid-derived suppressor cells, leukocytes, cytokines

## Abstract

Little information is available on the functional activity of leukocytes after arthroplasty or the expansion of populations with immune suppressive properties during the acute post-operative period. Synovial fluid and matched pre- and post-surgical blood samples were collected from total hip and knee arthroplasty patients (THA and TKA, respectively) to examine the impact of surgery on peripheral blood leukocyte frequency, bactericidal activity, and inflammatory mediator expression. For spinal surgeries, inflammatory mediator production by peripheral blood mononuclear cells (PBMCs) pre- and post-surgery was examined. An expansion of immune suppressive granulocytic myeloid-derived suppressor cells (G-MDSCs) was observed following arthroplasty, which correlated with significantly increased serum interleukin-10 (IL-10) levels. Analysis of synovial fluid from THA and TKAs revealed reduced granulocyte colony-stimulating factor (G-CSF) and soluble CD40 ligand (sCD40L) and increased interleukin-6 (IL-6), monocyte chemoattractant protein 2 (CCL2) and Fms-like tyrosine kinase 3 ligand (Flt-3L) compared to pre- and post-surgical serum. For the spinal surgery cohort, stimulation of PBMCs isolated post-surgery with bacterial antigens produced significantly less pro-inflammatory (IL-1α, IL-1β, interleukin-1 receptor antagonist (IL-1RA), IL-12p40, growth-related oncogene-α/GRO-α (CXCL1) and 6Ckine (CCL21)) and more anti-inflammatory/tissue repair mediators (IL-10, G-CSF and granulocyte-macrophage colony-stimulating factor (GM-CSF)) compared to PBMCs recovered before surgery. The observed bias towards systemic anti-inflammatory changes without concomitant increases in pro-inflammatory responses may influence susceptibility to infection following orthopaedic surgery in the context of underlying co-morbidities or risk factors.

## 1. Introduction

Joint arthroplasty is the most common orthopaedic procedure in the United States [1]. Total knee and hip arthroplasties (TKA and THA, respectively) alleviate pain and improve mobility and quality-of-life. Over 1 million procedures are performed in the United States each year [2] and by 2030, it is projected that the demand for primary and revision TKA and THA will total over 4 million cases [3,4]. This increase is driven by an aging population, higher rates of diagnosis and treatment of advanced arthritis, and expansion of surgical treatment to younger, more active patients [2]. Between 1998 and 2008, the annual number of spinal fusions in the United States increased from approximately 174,000 to over 400,000, in part, from improvements in instrumentation and surgical technique [5].

Surgical trauma can lead to alterations in hemodynamic, metabolic, and immune responses during the post-operative period [6]. A local inflammatory response in the surgical wound, typified by polymorphonuclear neutrophil (PMN) and monocyte recruitment, serves to limit tissue damage and remove cell debris to promote the healing process. Several studies have characterized this post-operative inflammatory reaction by the heightened production of several proinflammatory mediators, including interleukin (IL)-1β and tumor necrosis factor α (TNF-α), which can induce the release of other cytokines, such as IL-6 that has been correlated with post-operative complications after arthroplasty [7].

However, unlike reports of heightened pro-inflammatory responses post-surgery, other studies have shown that surgery induces immune suppression. For example, PMN, monocyte, and macrophage phagocytic activity is reduced during the post-operative period, providing a potential window for infection susceptibility [8,9,10]. We have shown that a heterogeneous subset of immature monocytes and granulocytes, called myeloid-derived suppressor cells (MDSCs), are recruited to tissues during prosthetic joint infection (PJI), where they exert anti-inflammatory effects [11,12,13]. However, to our knowledge, no studies have directly evaluated MDSC subsets after acute surgery in the orthopaedic setting, which may represent a potential biomarker to evaluate post-surgical inflammation. Increased levels of anti-inflammatory molecules, including IL-4, IL-10, soluble tumor necrosis factor receptor 1 (sTNFR1), IL-1 receptor antagonist (IL-1Ra), and transforming growth factor β (TGF-β) can also be produced during the post-operative period [14,15], which may further bias the anti-inflammatory attributes of wound-associated leukocytes.

Due to current discrepancies as to whether orthopaedic surgery elicits pro- vs. anti-inflammatory signatures, the objective of this study was to determine if a post-surgical immune signature exists that may explain, in part, why a subset of patients develop post-surgical infectious complications in the context of underlying co-morbidities or risk factors. To this end, we investigated pre- and post-operative changes in peripheral blood leukocyte populations and their activation status as well as inflammatory mediators in matched serum and synovial fluid samples in patients undergoing THA, TKA, and spinal procedures. These distinct orthopaedic procedures were compared to determine whether inflammatory signatures would be conserved, which would suggest that post-surgical inflammatory changes could play a broader role than previously appreciated.

## 2. Materials and Methods

### 2.1. Patient Population and Sample Procurement

Informed consent was obtained during the pre-surgical visit of patients undergoing primary THA and TKA or spine surgery. The demographics, surgery type, and diagnosis of subjects for both cohorts are provided in Table 1 and Table 2 (THA/TKA and spine surgery, respectively) with exclusion criteria in Table 3. The subjects reported in this manuscript represent a subset of two larger studies with objectives that were outside the scope of the immune parameters reported here. The spinal subjects were included in a study that enrolled 254 arthroplasty patients without synovial fluid collection (274 consented subjects, which included 33 spinal procedures, with 20 withdrawing from the study prior to surgery or for screen failures). The THA/TKA subjects were from a study that enrolled 114 patients with synovial fluid collection (145 consented subjects with 31 withdrawals for cancelled/rescheduled surgery, failure to obtain blood samples, or screen failure). Subjects who consented but had a negative synovial fluid sample (*n* = 3) were evaluated for pre and post changes in leukocyte frequency only. Samples for this study were collected from the larger studies at regular intervals without any bias to diagnosis. For all patients, venous blood was collected at the pre-surgical visit and again within 30 min post-surgery, during recovery. Synovial fluid was collected intraoperatively for THA and TKA only and was immediately transferred to a sterile specimen container and held on ice until processing. Both study protocols were approved by the Institutional Review Board of the University of Nebraska Medical Center (Omaha, NE, USA) (#177-14-FB and 792-16-EP).

### 2.2. Flow Cytometry

PBMCs from a subset of patients undergoing THA or TKA were analyzed by flow cytometry (*n* = 19). Within 60 min after each draw, blood was layered over Ficoll-Paque PLUS, leukocytes were collected from the interface, and remaining red blood cells (RBCs) were lysed using RBC Lysis Buffer (BioLegend; San Diego, CA, USA). After lysis, cells were washed, incubated with Human FcR Binding Inhibitor (eBioscience; San Diego, CA, USA), and stained with anti-human CD8a-AlexaFluor488, CD4-PE, CD66b-PE-Dazzle, CD25-APC, CD45-APC-Cy7, CD127-PerCP-Cy5.5, CD14-PE-Cy7, HLA-DR-BV421, CD15-BV510, CD19-BV605, CD33-BV711, and CD16-BV650 (all from BioLegend; San Diego, CA, USA). Dead cells were excluded using a LIVE/DEAD Fixable Blue Dead Cell Stain Kit (Life Technologies; Eugene, OR, USA) and analysis was performed using BD FACS-DIVA software, as previously described with the gating strategy depicted in Supplementary Figure S1 [16].

### 2.3. Multi-Analyte Microbead Array

Inflammatory mediator expression in samples was quantified using a MILLIPLEX MAP Human Cytokine/Chemokine Multiplex Assay (Cat. #HCYTOMAG-60K; Millipore, Billerica, MA, USA) according to the manufacturer’s instructions. Results were analyzed using a Bio-Plex Workstation (Bio-Rad, Hercules, CA, USA).

### 2.4. Whole Blood Killing Assay

*S. aureus* USA300 LAC 13c [17] was grown for 12 h in tryptic soy broth (250 rpm, 37 °C), washed twice in phosphate buffered saline (PBS), and diluted to 10⁶ colony forming units (CFU)/mL. A total of 100 µL of bacteria (10⁵ CFU) was mixed with 400 µL of freshly drawn blood in 5 mL fluorescence-activated cell sorting (FACS) tubes. Samples were incubated at 37 °C for 30, 60, and 120 min with constant agitation, whereupon dilutions were plated on trypticase soy agar with 5% sheep blood for enumeration of surviving CFU. In some experiments, blood was serially diluted to evaluate the possible action of interfering molecules (i.e., prozone phenomenon), which was not observed (Supplementary Figure S2).

### 2.5. Peripheral Blood Mononuclear Cell (PBMC) Stimulation

PBMCs were isolated within 60 min of each blood draw from patients before and after spinal surgery (*n* = 22) by Ficoll-Paque PLUS as described above, and cultured in a 96-well plate at 2 × 10⁵ cells/well in medium containing autologous serum. PBMCs were incubated for 1 h at 37 °C, 5% CO₂ before stimulation with *S. aureus* peptidoglycan (PGN; 2, 20, and 200 µg/mL), the synthetic triacylated lipopeptide Pam3CysSerLys4 (Pam3CSK4; 2, 20, and 200 µg/mL), or heat-killed *S. aureus* USA300 LAC 13c (10⁵, 10⁶, and 10⁷ CFU/mL) for 24 h, whereupon cell-free supernatants were collected and stored at −20 °C until assayed using the MILLIPLEX MAP Human Cytokine/Chemokine Multiplex Assay described above. Any value that was at least 2-fold higher than the unstimulated control was considered to be a positive response and assigned the following ranking: 0, no response at any concentration tested; 1 = positive response at the lowest dose tested (i.e., 2 µg/mL PGN/Pam3CSK4 or 10⁵ CFU heat-killed *S. aureus*); 2 = positive response at the middle dose tested (i.e., 20 µg/mL PGN/Pam3CSK4 or 10⁶ CFU heat-killed *S. aureus*); and 3 = positive response at the highest dose tested (i.e., 200 µg/mL PGN/Pam3CSK4 or 10⁷ CFU heat-killed *S. aureus*).

### 2.6. Statistics

For the PBMC analysis, a Wilcoxon signed rank test was used to compare the distribution of pre- and post-surgical responses. Significant differences in peripheral blood leukocyte populations pre- and post-arthroplasty were determined by an unpaired two-tailed Student’s *t*-test, while significant differences in Milliplex data between pre- and post-surgery sera and synovial fluid was determined by a one-way analysis of variance (ANOVA), using GraphPad Prism version 6 (La Jolla, CA, USA). For all analyses, a *p*-value of < 0.05 was considered statistically significant.

## 3. Results

### 3.1. Immune Alterations in the Peripheral Blood and Synovial Fluid of THA and TKA Patients

The first purpose of this study was to determine if orthopaedic procedures caused the expansion of MDSCs that possess immune inhibitory activity and whether this translated into differences in leukocyte effector function. Twenty-six patients were included in this study cohort, with the majority (~92%) requiring THA or TKA for osteoarthritis (OA) (Table 1). The average age was 63.4 years (range of 40–83) with 12 males and 14 females enrolled.

**Figure 1:**
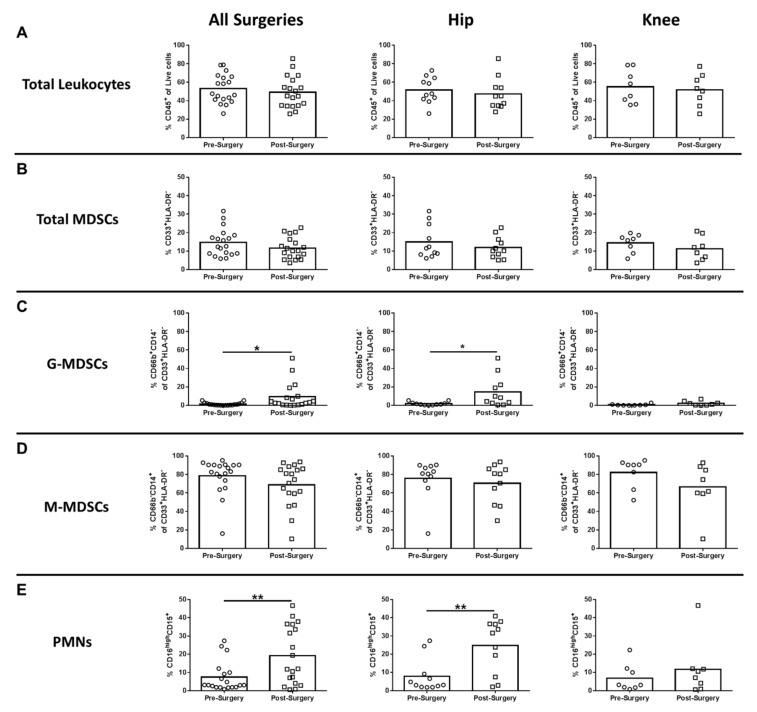
Hip/knee arthroplasty results in increased granulocytic myeloid-derived suppressor cells (G-MDSCs) and polymorphonuclear neutrophils (PMNs) in the peripheral circulation. Blood samples collected from patients pre- and post-surgery for total hip (THA) (*n* = 11) and total knee arthroplasties (TKA) (*n* = 8) were analyzed by flow cytometry. Results were calculated after gating on live CD45^+^ cells and quantitation of (**A**) total CD45^+^ leukocytes, (**B**) total myeloid-derived suppressor cells (MDSCs) (CD33^+^HLA-DR^-^), (**C**) G-MDSCs (% CD66b^+^CD14^-^ of the total MDSC population), (**D**) M-MDSCs (% CD66b^-^CD14^+^ of the total MDSC population), and (**E**) CD16^high^CD15^+^ neutrophils (PMNs) is reported (* *p* < 0.05; ** *p* < 0.01; unpaired two-tailed Student’s *t*-test).

Two MDSC subsets have been described, namely CD33^+^HLA-DR^-^CD66b^-^CD14^+^ monocytic-MDSCs (M-MDSCs) and CD33^+^HLA-DR^-^CD66b^+^CD14^low/-^ granulocytic-MDSCs (G-MDSCs) [18]. Although the percentages of total CD45^+^ leukocytes and MDSCs in the blood was not affected by surgery (Figure 1A,B, respectively), the granulocytic fraction of this population (G-MDSCs; CD66b^+^CD14^-^ of CD33^+^HLA-DR^-^) was significantly elevated following surgery for all arthroplasties and THA compared to pre-surgical matched controls (Figure 1C), whereas M-MDSCs were not affected (Figure 1D). PMNs (CD16^high^CD15^+^) were significantly increased after surgery in all procedures compared to pre-surgical matched controls, which was driven by THA patients (Figure 1E). Collectively, the observed increases in G-MDSCs and PMNs reflect a transition toward increased granulocytic populations in the blood post-arthroplasty.

**Figure 2:**
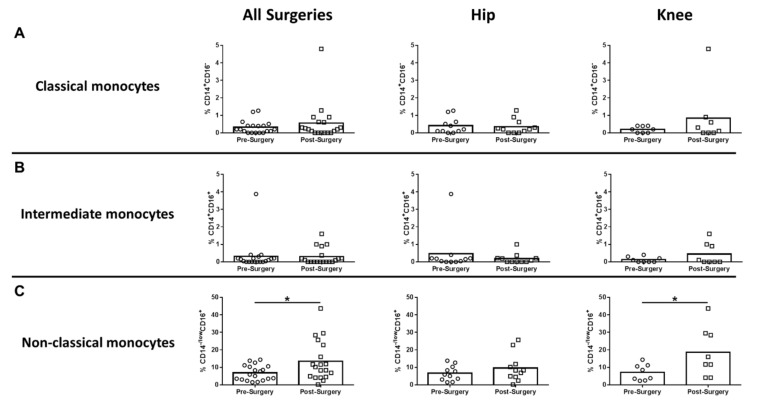
Non-classical monocytes are increased systemically following hip/knee arthroplasty. Blood samples collected from patients pre- and post-surgery for THA (*n* = 11) and TKA (*n* = 8) were analyzed by flow cytometry. Results were calculated after gating on live CD45^+^ cells and removing granulocyte and T cell populations from the analysis. Quantification of (**A**) CD14^+^CD16^-^ classical monocytes, (**B**) CD14^+^CD16^+^ intermediate monocytes, and (**C**) CD14^low^CD16^+^ non-classical monocytes is presented (* *p* < 0.05; unpaired two-tailed Student’s *t*-test).

**Figure 3:**
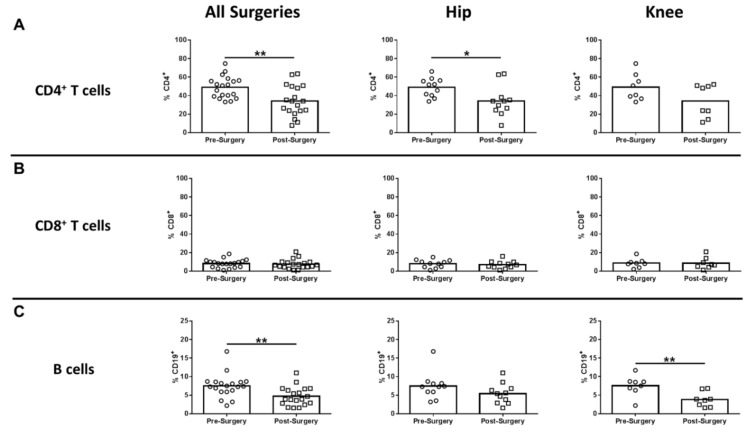
Hip/knee arthroplasty reduces CD4^+^ T and B cells in the peripheral circulation. Blood samples collected from patients pre- and post-surgery for THA (*n* = 11) and TKA (*n* = 8) were analyzed by flow cytometry. Results were calculated after gating on live CD45^+^ cells and quantitation of (**A**) CD4^+^ T cells, (**B**) CD8^+^ T cells, and (**C**) CD19^+^ B cells is presented (* *p* < 0.05; ** *p* < 0.01; unpaired two-tailed Student’s *t*-test).

In humans, monocytes comprise three subsets based on CD14 and CD16 expression, which include classical, intermediate, and non-classical monocytes [19,20]. Non-classical monocytes are considered the primary inflammatory subtype, whereas classical monocytes are phagocytic with limited inflammatory attributes. Intermediate monocytes comprise only a small percentage of circulating monocytes and are transitional, displaying both phagocytic and inflammatory function [19]. Only CD14^-/low^CD16^+^ non-classical monocytes were significantly increased after surgery compared to pre-surgical matched controls for all procedures, which was driven by TKA (Figure 2C). Both CD14^+^CD16^-^ classical and CD14^+^CD16^+^ intermediate monocytes were unchanged following surgery (Figure 2A,B). With regard to adaptive immunity, CD4^+^ cells decreased significantly for all surgeries and TKA compared to pre-surgical matched controls (Figure 3A), whereas CD8^+^ T cells were unaffected (Figure 3B). B cells were also significantly decreased post-surgery for all procedures versus pre-surgical matched controls, which was largely influenced by patients with TKA (Figure 3C).

**Figure 4:**
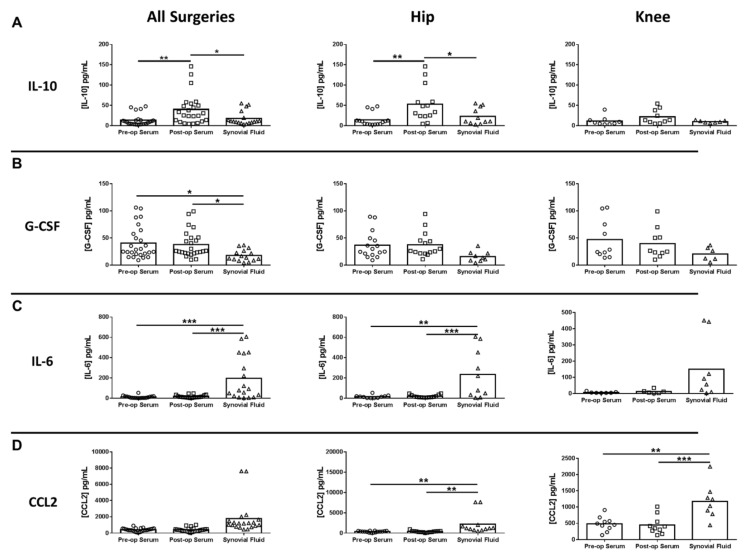
Cytokine and chemokine expression in serum and synovial fluid of hip/knee arthroplasty patients. Quantitation of (**A**) IL-10, (**B**) granulocyte colony-stimulating factor (G-CSF), (**C**) IL-6, and (**D**) monocyte chemoattractant protein 2 (CCL2) expression in pre- and post-sera and synovial fluid collected from THA (*n* = 16) and TKA (*n* = 10) subjects (* *p* < 0.05; ** *p* < 0.01; *** *p* < 0.001; one-way ANOVA).

**Figure 5:**
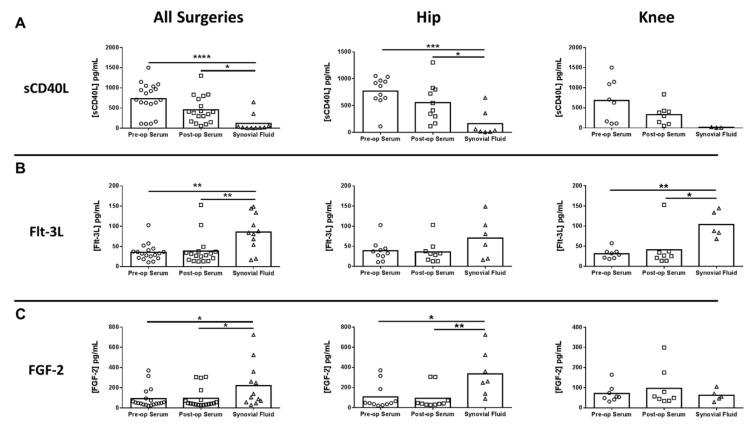
Differentially expressed mediators in the synovial fluid of hip/knee arthroplasty patients. Quantitation of (**A**) soluble CD40 ligand (sCD40L), (**B**) Fms-like tyrosine kinase 3 ligand (Flt-3L), and (**C**) basic fibroblast growth factor (FGF-2) expression in pre- and post-sera and synovial fluid collected from THA (*n* = 11) and TKA (*n* = 8) subjects (* *p* < 0.05; ** *p* < 0.01; *** *p* < 0.001; **** *p* < 0.0001; one-way ANOVA).

Next, inflammatory mediator expression was quantified in pre- and post-surgical sera as well as synovial fluid in THA/TKA patients to identify novel biomarkers that might serve a prognostic value. Of the mediators examined, only serum IL-10 was significantly increased in all procedures post-surgery, which was driven by THA (Figure 4A). In contrast, several mediators were significantly altered in synovial fluid versus pre- or post-surgical sera. For example, granulocyte-colony stimulating factor (G-CSF) was significantly decreased in the synovial fluid for all procedures compared to pre- and post-surgical sera (Figure 4B). Conversely, both IL-6 and monocyte chemoattractant protein 2 (CCL2) were increased in the synovial fluid of THA and TKA compared with pre- and post-surgical sera (Figure 4C,D, respectively), demonstrating the compartmentalization of inflammatory responses. In addition to IL-6 and CCL2, three additional mediators were differentially expressed in the synovial fluid, namely soluble CD40 ligand (sCD40L), Fms-like tyrosine kinase 3 ligand (Flt-3L), and basic fibroblast growth factor (FGF-2) (Figure 5).

**Figure 6:**
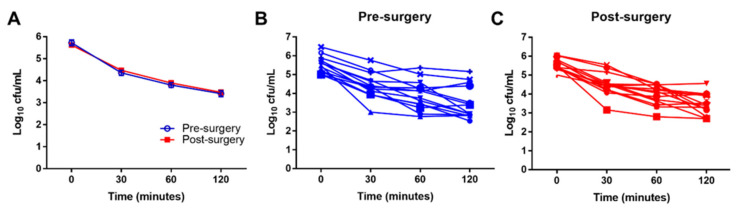
Surgery does not impair leukocyte bactericidal activity. *S. aureus* whole blood killing assays were performed from blood collected from patients pre- and post-surgery for THA and TKA (*n* = 14). Numbers of viable bacteria are reported as Log_10_ colony forming units (cfu) per mL for (**A**) the average killing for all patients pre- and post-arthroplasty and stratified into (**B**) pre-surgery and (**C**) post-surgery.

To investigate whether leukocyte function may be altered after surgery, *S. aureus* killing assays were performed on whole blood collected from THA and TKA patients. Although bacterial killing was more robust in some patients, overall pre- or post-surgical status had no effect on *S. aureus* bactericidal activity (Figure 6).

### 3.2. Effect of Spinal Arthroplasty on PBMC Responsiveness

Another purpose of this study was to determine whether orthopaedic surgery altered the activation state of PBMCs. For this analysis, a separate cohort of 22 spinal surgery patients was examined (Table 2), where PBMC responses to *S. aureus*-relevant stimuli were tested. The average age of subjects was 66.5 years (range of 38–87) with 12 males and 10 females enrolled in the study. Due to variation in the absolute values of mediators between patients, data were categorized based on at least a 2-fold-change in each mediator compared to unstimulated PBMCs from the same patient. Post-surgery, PBMCs had reduced expression of several pro-inflammatory mediators, including IL-12p40, IL-1β, IL-1α, and 6Ckine (CCL21) compared to pre-surgical matched controls (Table 4). In contrast, the anti-inflammatory cytokine IL-10 and growth factors (G-CSF and GM-CSF) were increased post-arthroplasty versus the pre-surgical control group (Table 5). To our knowledge, these findings are the first to report that surgery biases leukocytes towards a functional anti-inflammatory phenotype, in agreement with the post-surgical elevation in serum IL-10 in the THA/TKA cohort.

## 4. Discussion

Currently, some discrepancies exist as to whether orthopaedic surgery elicits pro- versus anti-inflammatory changes. Although prior studies have reported cytokine alterations following THA/TKA [21,22,23,24,25], it is paramount to address what effect this post-surgical immune response has on leukocyte function, which has received less attention. We were also interested in determining whether inflammatory signatures would be conserved across distinct orthopaedic procedures, which led to the investigation of both spinal procedures and THA/TKA. Our findings that anti-inflammatory responses are elicited following surgery in both orthopaedic settings suggests that post-surgical inflammatory skewing could play a broader role than previously appreciated. The anti-inflammatory bias reported in this study following orthopaedic surgeries may be a contributing, but not causative, factor for infectious complications, since the low incidence of PJI implies the involvement of other co-morbidities or risk factors [26,27,28,29].

Orthopaedic surgery induced the expansion of G-MDSCs that potently inhibit T cell proliferation [30,31], in agreement with the significant reduction in CD4^+^ T cells post-arthroplasty compared to matched pre-surgical samples. The expansion of G-MDSCs post-arthroplasty coincided with increased serum G-CSF compared to synovial fluid, a critical growth factor for MDSC and PMN expansion/differentiation [32,33]. In addition, PBMCs exposed to *S. aureus*-relevant stimuli produced significantly more G-CSF and GM-CSF after spinal procedures, suggesting that surgery primes leukocytes for increased growth factor production. Our findings of PMN expansion and CD4^+^ T cell contraction after arthroplasty are in agreement with prior studies that reported increased neutrophil-to-lymphocyte ratios in TKA and THA patients during the acute post-surgical period [14,34,35,36,37,38].

IL-10 was increased in both THA/TKA and spine patients, which may enhance patient susceptibility to infectious complications during the post-operative period in combination with other underlying risk factors or co-morbidities. Indeed, our recent study reported that IL-10 levels were significantly increased in patients with PJI compared to aseptic loosening [16] and in a mouse PJI model IL-10 was critical for promoting *S. aureus* persistence, in part, via inhibition of monocyte/macrophage pro-inflammatory activity that was mediated by MDSCs [11]. Elevated IL-10 levels have also been associated with impaired long-term functional performance following TKA [39] as well as in patients with traumatic bone injuries, which was predictive of sepsis development [40].

Flt-3L, a cytokine that promotes hematopoietic cell proliferation [41], was identified as a potential novel biomarker for OA, since its expression was significantly increased in the synovial fluid of arthroplasty patients. CCL2 levels were also increased in the synovial fluid, in agreement with an earlier report in patients with knee arthroscopy for cartilage tears [42]. IL-6 is a pro-inflammatory cytokine involved in many physiological and pathological processes [43,44], including MDSC expansion and activation [45]. IL-6 levels were increased in the synovial fluid of THA/TKA patients, where approximately 92% of these subjects were diagnosed with OA, in agreement with prior reports [44,46,47]. The overall lack of pro-inflammatory mediators in the serum after surgery in our study differs from earlier reports describing elevations in IL-6, TNF-α, and IL-1β following THA or TKA [21,22,23,24,25]. The reasons for this discrepancy remain unknown, but could be explained by the exclusion criteria for our study, since consented subjects had fewer underlying conditions that may potentiate pro-inflammatory reactions after surgery. We acknowledge the limitations of our study, including the cohort size and lack of serial sampling. However, prior studies have reported that many inflammatory changes after THA/TKA manifest during the acute post-surgical period [22,34,36], similar to the interval examined here.

This report advances the field by revealing that arthroplasty induces changes in leukocyte activation status. This is reflected by an overall decrease in pro-inflammatory mediator expression by PBMCs post-surgery concomitant with increased anti-inflammatory (IL-10) and growth factor production, molecules that are typically associated with wound healing. The finding that anti-inflammatory responses are elicited following diverse orthopaedic procedures (i.e., spinal surgery and THA/TKA) suggests that post-surgical inflammatory skewing could play a broader role than previously appreciated. Future longitudinal studies should be performed to examine the potential of peripheral blood G-MDSCs as a biomarker for the risk of developing PJI or monitoring the extent of post-surgical inflammation.

## Figures and Tables

**Table 1:** Hip and knee arthroplasty subjects.

Age	Sex	Surgery Type	Diagnosis
68	M	Primary Hip	Osteoarthritis
76	M	Primary Hip	Osteoarthritis
58	F	Primary Hip	Osteoarthritis
83	F	Primary Hip	Osteoarthritis
53	F	Primary Hip	Osteoarthritis
66	F	Primary Hip	Osteoarthritis
48	M	Primary Knee	Osteoarthritis
40	M	Primary Hip	Osteoarthritis
70	M	Primary Knee	Trauma
68	M	Primary Knee	Osteoarthritis
64	F	Primary Knee	Osteoarthritis
63	F	Primary Hip	Osteoarthritis
49	F	Primary Hip	Osteoarthritis
77	F	Primary Knee	Osteoarthritis
65	F	Primary Knee	Osteoarthritis
65	F	Primary Knee	Osteoarthritis
64	M	Primary Hip	Osteoarthritis
79	M	Primary Hip	Osteoarthritis
68	M	Primary Hip	Osteoarthritis
67	F	Primary Hip	Traumatic arthritis
48	M	Primary Hip	Avascular Necrosis
62	F	Primary Knee	Osteoarthritis
69	F	Primary Hip	Osteoarthritis
42	M	Primary Hip	Osteoarthritis
72	M	Primary Knee	Osteoarthritis
65	F	Primary Knee	Osteoarthritis

**Table 2:** Spinal procedure subjects.

Age	Sex	Surgery Type	Diagnosis
70	M	Primary	Stenosis
72	M	Primary	Anterior cervical discectomy with fusion
69	M	Revision	Osteoarthritis
57	M	Primary	Stenosis
63	F	Primary	Stenosis, spondylolisthesis
62	F	Primary	Stenosis, spondylolisthesis
57	M	Primary	Osteoarthritis
75	F	Primary	Stenosis, spondylolisthesis
70	M	Primary	Stenosis, Scoliosis/Kyphoscoliosis
66	F	Primary	Stenosis, myelopathy, kyphosis
70	F	Primary	Stenosis, spondylolisthesis
77	M	Primary	Stenosis, spondylolisthesis
75	M	Primary	Stenosis, spondylolisthesis
38	F	Revision	Cervical stenosis and DDD *
57	M	Primary	Cervical spondylosis
74	M	Primary	Stenosis, spondylolisthesis, arthritis
73	M	Revision	Loosening, recurrent stenosis
68	F	Primary	Stenosis, spondylolisthesis
64	F	Primary	Stenosis, spondylolisthesis
56	F	Primary	Cervical DDD and stenosis
62	F	Primary	Stenosis
87	M	Primary	Stenosis

* Degenerative disc disease.

**Table 3:** Study exclusion criteria.

Criteria	Description
1	Immunocompromised
2	Diagnosed with a known bleeding disorder
3	Received blood products 60 days prior to consent
4	History of leukemia, lymphoma, or underlying bone marrow disorder
5	Malignancy that required treatment with chemotherapy (including the use of adjunctive and hormonal therapy), immunotherapy, radiation therapy, or other antineoplastic target therapy within the past 24 months
6	Congenital, functional, or surgical asplenia
7	End stage renal disease (defined as requiring or anticipating requirements for hemodialysis, peritoneal dialysis, or renal transplant) or nephrotic syndrome
8	Previous administration of *S. aureus* or *S. aureus*/Candida vaccine or *S. aureus* immunoglobulins (monoclonal or polyclonal)
9	Participation in other studies involving investigational drug(s) (Phases 1-4) within 30 days prior to consent and/or during study participation

**Table 4:** Mediators decreased in PBMCs following spinal arthroplasty.

Mediator	Variable	*N*	Median	Min	Max	*p*-Value
IL-12p40 *	Pre-PGN	22	3	0	3	
	Post-PGN	21	0	0	3	
	PGN (Post-Pre)	21	−1	−3	1	0.004
	Pre-Pam3CSK4	21	3	0	3	
	Post-Pam3CSK4	19	0	0	3	
	Pam3CSK4 (Post-Pre)	18	−1	−3	0	0.002
	Pre-HKSA	22	2	0	3	
	Post-HKSA	21	0	0	3	
	HKSA (Post-Pre)	21	−1	−3	1	0.003
**IL-1** **β**	Pre-PGN	21	3	2	3	
	Post-PGN	21	2	0	3	
	PGN (Post-Pre)	20	0	−3	1	0.1133
	Pre-Pam3CSK4	21	3	0	3	
	Post-Pam3CSK4	19	2	0	3	
	Pam3CSK4 (Post-Pre)	18	0	−3	1	0.0449
	Pre-HKSA	22	2	1	3	
	Post-HKSA	20	2	0	3	
	HKSA (Post-Pre)	20	−1	−3	1	0.0041
**IL-1** **α**	Pre-PGN	21	2	0	3	
	Post-PGN	20	2	0	3	
	PGN (Post-Pre)	20	0	−3	2	0.125
	Pre-Pam3CSK4	20	3	1	3	
	Post-Pam3CSK4	18	2	0	3	
	Pam3CSK4 (Post-Pre)	17	−1	−3	1	0.014
	Pre-HKSA	21	2	0	3	
	Post-HKSA	20	2	0	3	
	HKSA (Post-Pre)	20	0	−2	1	0.036
**CCL21**	Pre-PGN	22	0	0	3	
	Post-PGN	21	0	0	2	
	PGN (Post-Pre)	21	0	−3	2	0.023
	Pre-Pam3CSK4	21	2	0	3	
	Post-Pam3CSK4	20	0.5	0	3	
	Pam3CSK4 (Post-Pre)	19	−1	−3	3	0.075
	Pre-HKSA	22	0.5	0	3	
	Post-HKSA	21	0	0	2	
	HKSA (Post-Pre)	21	0	−3	1	0.006

* IL-12: interleukin-12; PGN: peptidoglycan; Pam3CSK4: synthetic triacylated lipopeptide Pam3CysSerLys4; HKSA: heat-killed *S. aureus*, CCL21: C-C chemokine 21/6Ckine.

**Table 5:** Mediators increased in PBMCs following spinal arthroplasty.

Mediator	Variable	*N*	Median	Min	Max	*p*-Value
G-CSF *	Pre-PGN	22	3	1	3	
	Post-PGN	21	2	0	3	
	PGN (Post-Pre)	21	−1	−3	1	0.0093
	Pre-Pam3CSK4	21	3	1	3	
	Post-Pam3CSK4	19	2	0	3	
	Pam3CSK4 (Post-Pre)	18	−0.5	−3	2	0.0273
	Pre-HKSA	22	2	2	3	
	Post-HKSA	21	2	0	3	
	HKSA (Post-Pre)	21	0	−2	1	0.1023
**GM-CSF**	Pre-PGN	22	3	0	3	
	Post-PGN	21	1	0	3	
	PGN (Post-Pre)	21	−1	−3	1	0.0002
	Pre-Pam3CSK4	21	3	0	3	
	Post-Pam3CSK4	19	1	0	3	
	Pam3CSK4 (Post-Pre)	18	−1.5	−3	1	0.0013
	Pre-HKSA	22	2	0	3	
	Post-HKSA	21	2	0	3	
	HKSA (Post-Pre)	21	0	−3	2	0.3008
**IL-10**	Pre-PGN	22	3	0	3	
	Post-PGN	21	2	0	3	
	PGN (Post-Pre)	21	0	−3	1	0.012
	Pre-Pam3CSK4	21	3	1	3	
	Post-Pam3CSK4	19	2	0	3	
	Pam3CSK4 (Post-Pre)	18	0	−3	1	0.023
	Pre-HKSA	22	3	2	3	
	Post-HKSA	21	2	0	3	
	HKSA (Post-Pre)	21	0	−3	1	0.022

* G-CSF: granulocyte colony-stimulating factor; GM-CSF: granulocyte-macrophage colony-stimulating factor; IL-10: interleukin-10; PGN: peptidoglycan; Pam3CSK4: synthetic triacylated lipopeptide Pam3CysSerLys4; HKSA: heat-killed *S. aureus*.

## Supplementary Materials

The following are available online at https://www.mdpi.com/2077-0383/9/7/2123/s1, Figure S1: Gating strategy to quantitate leukocyte populations in whole blood. Single cells were gated from the total events using FSC-A vs. FSC-H, followed by exclusion of dead cells. Live, CD45+ leukocytes were separated into granulocytes (CD16^high^CD15^+^) and non- granulocytes. Non-granulocyte populations were identified using a series of gates to avoid duplicate counting. First, MDSCs were identified as CD33^+^HLA-DR^-^ cells, followed by CD4^+^ and CD8^+^ T cell populations out of the non-MDSC population. CD19 was used to identify B cells within the non-T cell population and finally, the abundance of monocyte populations was determined using CD14 and CD16 expression, Figure S2: *S. aureus* whole blood killing assay is not influenced by prozone phenomenon. Matched pre-/post-arthroplasty blood samples were collected from a patient and serially diluted to determine whether inhibitory factors influenced *S. aureus* killing. Results are representative of findings from two individual patients.
