# Supplementary material for: Orthopaedic Surgery Elicits a Systemic Anti-Inflammatory Signature
Source: J Clin Med. 2020 Jul 6;9(7):2123. doi: 10.3390/jcm9072123 (PMC7408679; doi:10.3390/jcm9072123)
Supplement: Supplementary file 1 [file jcm-09-02123-s001.pdf]

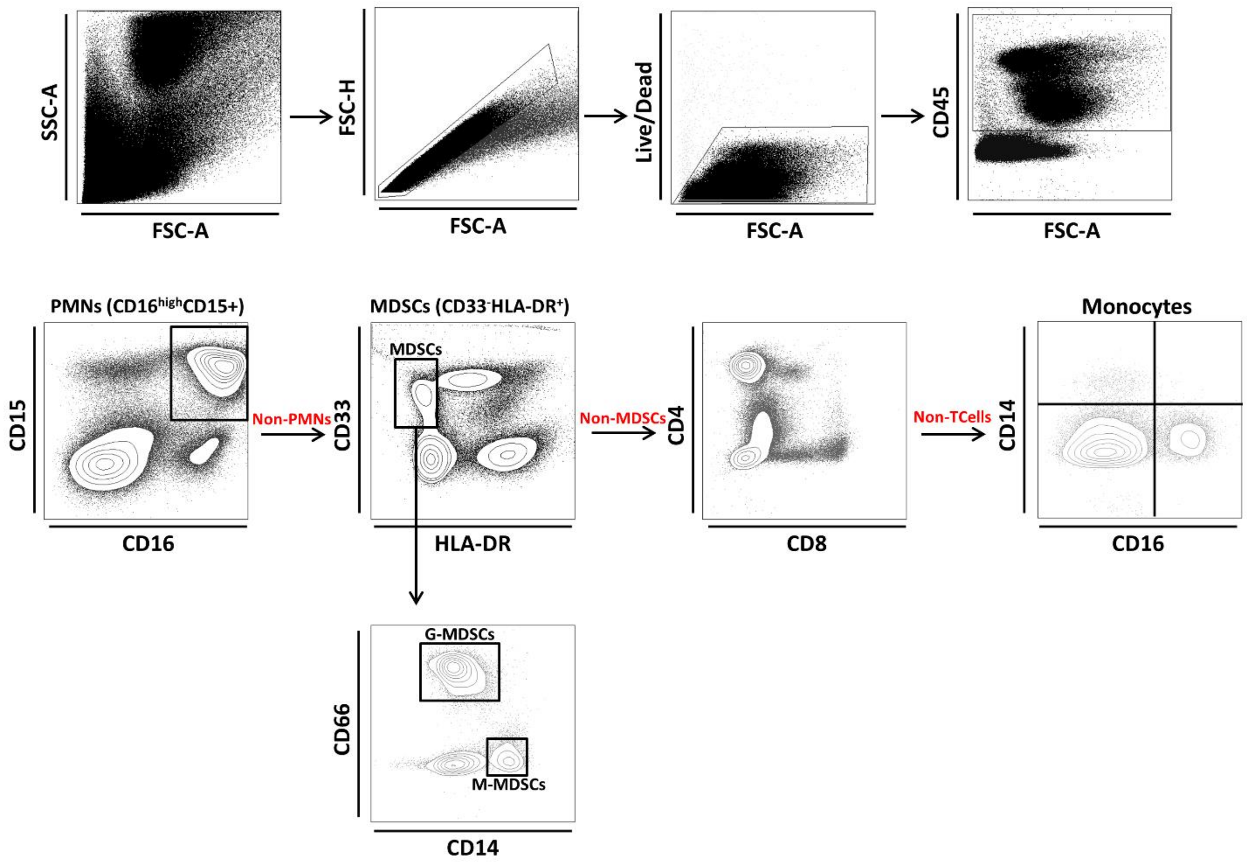

Supplemental Figure 1. Gating strategy to quantitate leukocyte populations in whole blood. Single cells were gated from the total events using FSC-A vs. FSC-H, followed by exclusion of dead cells. Live, $CD45^+$ leukocytes were separated into granulocytes ($CD16^{high}CD15^+$) and non-granulocytes. Non-granulocyte populations were identified using a series of gates to avoid duplicate counting. First, MDSCs were identified as $CD33^+HLA\text{-}DR^-$ cells, followed by $CD4^+$ and $CD8^+$ T cell populations out of the non-MDSC population. CD19 was used to identify B cells within the non-T cell population and finally, the abundance of monocyte populations was determined using CD14 and CD16 expression.

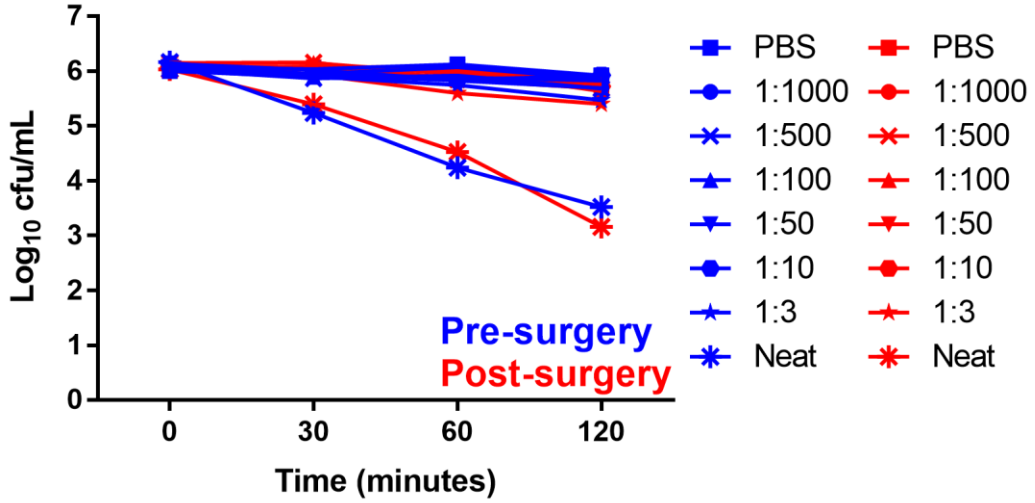

Supplemental Figure 2. *S. aureus* whole blood killing assay is not influenced by prozone phenomenon. Matched pre-/post-arthroplasty blood samples were collected from a patient and serially diluted to determine whether inhibitory factors influenced *S. aureus* killing. Results are representative of findings from two individual patients.
